# Deep Sequencing of the Nicastrin Gene in Pooled DNA, the Identification of Genetic Variants That Affect Risk of Alzheimer's Disease

**DOI:** 10.1371/journal.pone.0017298

**Published:** 2011-02-25

**Authors:** Michelle K. Lupton, Petroula Proitsi, Makrina Danillidou, Magda Tsolaki, Gillian Hamilton, Richard Wroe, Megan Pritchard, Kathryn Lord, Belinda M. Martin, Iwona Kloszewska, Hilkka Soininen, Patrizia Mecocci, Bruno Vellas, Denise Harold, Paul Hollingworth, Simon Lovestone, John F. Powell

**Affiliations:** 1 MRC Centre for Neurodegeneration Research, Institute of Psychiatry, King's College London, London, United Kingdom; 2 3rd Department of Neurology, Aristotle University of Thessaloniki, Thessaloniki, Greece; 3 Medical Genetics, Molecular Medicine Centre, Western General Hospital, University of Edinburgh, Edinburgh, United Kingdom; 4 Department of Old Age Psychiatry and Psychotic Disorders, Medical University of Lodz, Lodz, Poland; 5 Department of Neurology, University of Eastern Finland and Kuopio University Hospital, Kuopio, Finland; 6 Section of Gerontology and Geriatrics, Department of Clinical and Experimental Medicine, University of Perugia, Perugia, Italy; 7 Department of Internal and Geriatrics Medicine, Hôpitaux de Toulouse, Toulouse, France; 8 Department of Psychological Medicine and Neurology, MRC Centre for Neuropsychiatric Genetics and Genomics, School of Medicine, Cardiff University, Cardiff, United Kingdom; King's College London, United Kingdom

## Abstract

Nicastrin is an obligatory component of the γ-secretase; the enzyme complex that leads to the production of Aβ fragments critically central to the pathogenesis of Alzheimer's disease (AD). Analyses of the effects of common variation in this gene on risk for late onset AD have been inconclusive. We investigated the effect of rare variation in the coding regions of the Nicastrin gene in a cohort of AD patients and matched controls using an innovative pooling approach and next generation sequencing. Five SNPs were identified and validated by individual genotyping from 311 cases and 360 controls. Association analysis identified a non-synonymous rare SNP (N417Y) with a statistically higher frequency in cases compared to controls in the Greek population (OR 3.994, CI 1.105–14.439, p = 0.035). This finding warrants further investigation in a larger cohort and adds weight to the hypothesis that rare variation explains some of genetic heritability still to be identified in Alzheimer's disease.

## Introduction

Alzheimer's disease (AD) is the commonest neurodegenerative disease of the elderly and with an increasingly aging population in many countries the burden of disease will continue to rise inexorably. Until recently, the ε4 allele of the APOE gene was the only well established genetic risk factor for the typical late-onset form of the disease. However, recent results from two of the largest genome wide association studies have identified and replicated several novel loci with high significance [1], [2]. These loci include clusterin (CLU) and complement receptor 1 (CR1). In contrast to the APOE locus, the effect size and attributable risk for these variants are more modest. For example the CR1 locus has odds ratios of 1.11 and attributable fractions of 3.8% compared to 25.5% for APOE [2]. It is clear from these studies that in common with other complex disorders such as Type II diabetes or physiological traits such as height that susceptibility genes detected by whole genome association studies for AD are common (MAF>0.1) and of modest effect (RR<1.3, with APOE being a notable exception). Cumulatively these variants only explain a small proportion of the genetic risk; additional risk will undoubtedly be explained by more common variants of small effect. However, it is increasingly recognised that rare variants have a role in complex disease. An example in neurodegenerative disease is the glucocerebrosidase gene (GBA) in Parkinson's disease, individuals homozygous for mutations in this gene present with the Mendelian disorder Gaucher disease, while individuals heterozygous for the same mutations have an increased risk for Parkinson disease [3], [4] The cumulative frequency of GBA mutations in Parkinson disease cases can be as high as 9.0% compared to less than 0.5% in controls. Such variants are not included on commercial genotyping arrays but are only detected through re-sequencing approaches.

While genome wide association studies are appropriately powered to detect common variants of modest effect, linkage studies are more effective in detecting rare variants of larger effect even in the presence of allelic heterogeneity. Linkage studies in AD may therefore indicate loci harbouring rare variants and nominate candidate genes for re-sequencing. In a meta-analysis of AD linkage studies by Butler et al, 2009 [5], although there were no genome wide significant findings, three regions showed genome wide suggestive evidence for linkage. The gene for Nicastrin, one of the components of the γ-secretase is underneath one of the suggestive linkage peaks on chromosome 1.

Intramembranous γ-secretase cleavage of the amyloid precursor protein (APP) is the final step in the production of the amyloid-β peptide (Aβ); the main constituent of amyloid plaques found in the brains of patients with Alzheimer's disease [6]. The length of the Aβ peptide is dependent on the γ-secretase cleavage site, with Aβ42 being the most amyloidogenic peptide. Mutations in another component of the γ-secretase, presenilin 1 (PS1) are found in the early onset autosomal dominant form of Alzheimer's disease [7], [8]. Nicastrin was first identified by immunoprecipitation of PS1 followed by analysis by mass spectrometry and it acts as the substrate binding component of the γ-secretase complex [9].

Several studies have investigated the role of common SNPs in the Nicastrin gene on risk of AD. Haplotype studies have identified a haplotype (HapB) associated with early onset AD in people who lack the APOE ε4 risk variant in a Dutch population based sample [10]. This association was replicated in a Sardinian study containing both familial and sporadic AD cases, but failed to be significantly replicated in others [11], [12]. A study looking at normative cognitive aging found an effect of HapB on life long stable cognitive ability, but not on age related cognitive change [13]. There have also been investigations of the effect of Nicastrin promoter SNPs but again with varying results in different populations [13]–[16].

Systematic screens for rare variants that may have an effect on protein function have been carried out using small sample sizes comprising mostly familial AD cases. Dermaut et al (2002) [10] screened 78 familial and 38 sporadic cases using DHPLC indentifying 4 SNPs in the coding regions. One of which caused an amino acid substitution (N to Y at codon 417). Confalaoni et al (2003) [17] also identified this as the only missense mutation, present in 1 of 104 familial cases, 2 of 174 sporadic cases and one of 191 controls (although a young control). These studies use a combination of familial and sporadic AD cases which may have a heterogeneous genetic aetiology.

In addition non-sense and truncating mutations in the genes of the γ-secretase complex, including Nicastrin have recently been implicated in Familial Acne Inversa and haploinsufficency was inferred to be causal. None of the mutations have previously been shown to cause early onset AD, but it may be that these could contribute to risk of late onset AD [18].

To investigate the effect of rare variation in a larger sample size of sporadic AD patients we have re-sequenced the coding regions of the Nicastrin gene in a cohort of AD patients and matched controls using an innovative pooling approach and next generation sequencing.

## Results

Genomic DNA from 311 Alzheimer's patients and 360 controls were combined to create case and control pools. Nicastrin exon regions were amplified using 16 exon-specific PCR reactions, and then PCR products were pooled in equimolar amounts and subjected to deep sequencing.

**Figure 1:**
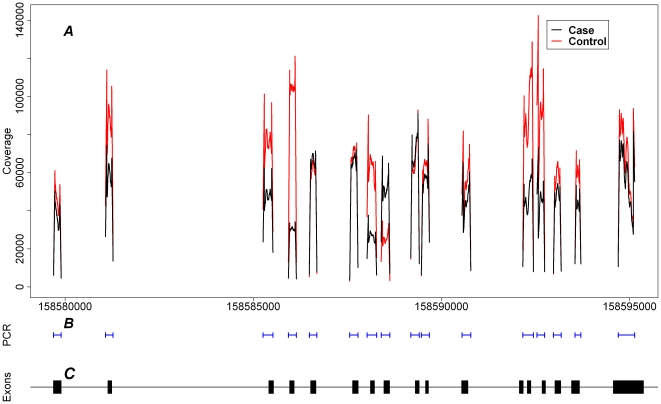
Depth of coverage. A: The depth of coverage at each position where the X axis shows the genomic position on chromosome 1 (hg18, Mar. 2006). B: Locations of the PCR products aligned to the X axis. C: Locations of the Exons of Nicastrin aligned to the X axis.

The sequencing of the case and control pools generated 307005 and 302171 reads respectively. For the case and control pools 87.1% and 87.0% of reads respectively mapped to the reference sequence. This resulted in a mean coverage of 228 reads per position for each individual in the case pool (114 per allele) and 197 reads per position for each individual in the control pool (98 reads per allele). Fig. 1A shows the distribution of read coverage. Using the cut off for variant calling described in the methods we were able to identify SNPs at a frequency of 3–8 variants in the pool, depending on the transition type. For example in the case pool the error distribution allowed the identification of a G-A transversion with a frequency of 3 variants, but for a A-C transition the error distribution was higher only allowing the identification of variants with a frequency as low as 7.

Table 1 shows the identified variants and those which were individually genotyped using Sequenom genotyping, for the purpose of validation. These included all common SNPs, all non-synonymous SNPs and a selection of the remaining rare SNPs identified. The SNPs at positions 158585487 and 158594972 had a frequency below the cut off for variant calling, but these were investigated in addition to the called SNPs (SNP 158585487 if validated would cause a non-synonymous change that would be potentially damaging, and SNP 158594972 is at a very high frequency only in one read direction). The common SNP at position 158587689 was not genotyped as it is known to be in perfect LD with the genotyped SNP at position 158588123 [17].

**Table 1:** Identified variants indicating the frequency in the pool and the individual genotyping results where carried out.

Position (hg18, Mar. 2006)	Reference Base	Base Change	Amino Acid Change	Sequencing Results						Individual Genotyping Results	
				Case Pool			Control Pool			CaseFreq (N)	ControlFreq (N)
				Freq	Depth	F∶R ratio score*	Freq	Depth	F∶R ratio score*		
158579744	T	G		0.011	43865	0.06	0.012	53337	0.35	0.000 (290)	0.000 (343)
158579822	A	G	4A>A	0.008	29914	0.75	0	37900	Not present		
158579842	A	G	11D>G	0.034	32766	1.09	0.033	40522	1.05	0.000 (290)	0.000 (342)
158585459	G	A	79E>E	0.015	49008	0.41	0	77896	Not present		
158585487	G	T**	89G>C	0.009	62187	0.10	0.009	96747	0.10	0.000 (290)	0.000 (345)
158587689	A	G	212L>L	0.038	68900	0.10	0.054	72522	0.19		
158588079	G	A		0.006	24984	0.54	0	59781	Not present		
158588123	C	T	249D>D	0.033	28255	0.32	0.054	67365	0.40	0.035 (276)	0.048 (344)
158588214	A	T	280T>S	0.006	23823	1.68	0.006	54929	0.14	0.000 (282)	0.000 (339)
158588214	A	C	280T>P	0.093	23823	1.68	0.100	54929	0.14	0.002 (280)	0.000 (341)
158590601	A	T	417N>Y	0.012	33904	0.77	0	41705	Not present	0.019 (287)	0.004 (345)
158592193	T	G		0.016	49363	0.14	0.017	91556	0.22	0.002 (288)	0.000 (345)
158592559	T	C		0.013	61645	0.44	0.014	119627	0.42	0.000 (290)	0.000 (343)
158593669	T	G		0.009	51485	0.37	0	66754	Not present		
158594855	G	C		0	61686	Not present	0.005	72880	0.42		
158594885	A	C		0.014	51872	0.95	0	60335	Not present	0.000 (290)	0.000 (343)
158594972	G	T**		0.042	49352	1.56	0.051	58461	1.62	0.000 (288)	0.001 (345)
158594975	A	C		0.026	47088	0.81	0.027	55505	0.83		
158594975	A	T		0	47088	Not present	0.012	55505	1.29198185		

*The forward to reverse ratio score is the absolute difference between the frequencies in the forward and reverse reads divided by the mean frequency.

**indicates SNPs that did not pass the threshold for SNP calling but were still tested for validation.

Five SNPs identified in the sequencing were validated by individual genotyping. In addition the two SNPs that did not pass the acceptable threshold that were genotyped were not validated. These are likely to be due to sequencing or alignment errors. Most of the SNPs with a minor allele frequency below 0.01 were false positive although two rare variants were validated at a low frequency. Of the common variants identified only the SNP at position 158588123 was validated, which also validates the SNP at position 158587689. Three SNPs that had relatively high MAF (0.03–0.1) were not validated in the single genotyping. On closer inspection it can be seen that these SNPs have an extremely high forward to reverse read ratio, being overly represented in the forward reads possibly indicative of a sequencing artefact. The level of discrepancy between forward and reverse reads was represented using a score which was calculated from the absolute difference between the frequencies in the forward and reverse reads divided by the mean frequency (table 1).

**Figure 2:**
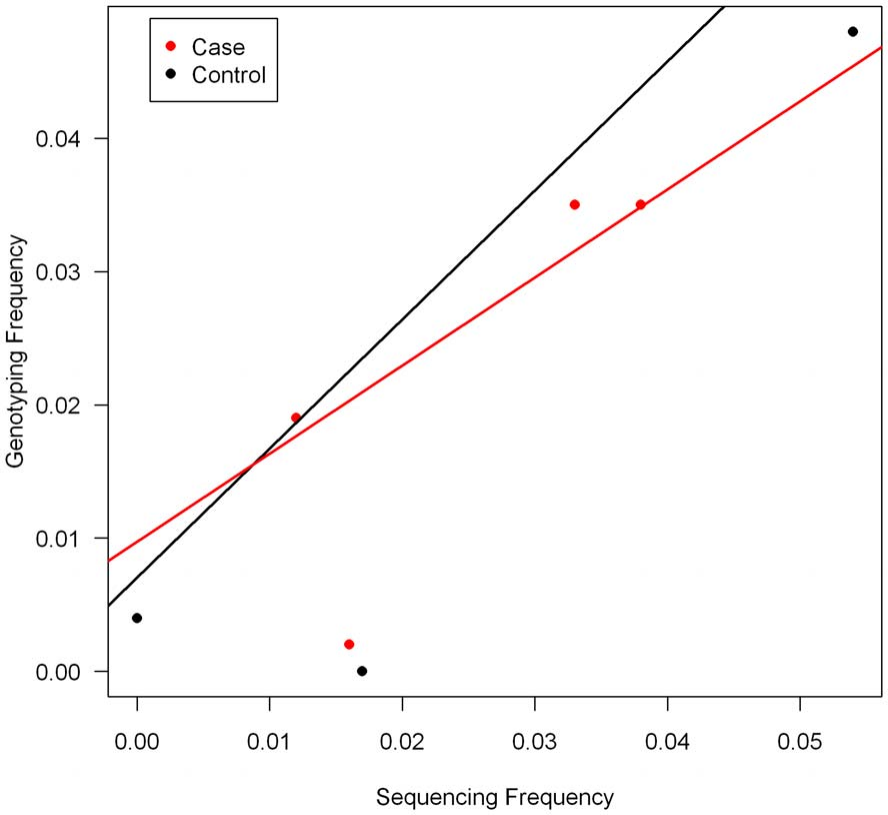
Comparison of allele frequencies for SNPs identified by sequencing and validated by individual genotyping. Only SNPs with a ratio score of 1 or lower were considered (see text for details).

The allele frequencies for validated SNPs were compared between SNPs with a F∶R read ratio score of 1 or lower (fig. 2). The correlation between the Sequencing in pools and the individual genotyping is 0.82 and 0.95 for case and control respectively. If the outlier (SNP at position 158592193 which is in 32 bp from the start of the PCR product and in a relatively low depth region) is excluded the correlation becomes stronger, 0.98 for cases and 1 for controls.

Association analysis was carried out on the variants validated by individual genotyping (table 2). No association with AD was identified for the common SNP at position 158588123. This is located in exon 10 and results in a synonymous change. Of the rare variants identified, two resulted in non-synonymous amino acid changes. Both were predicted to be possibly damaging using POLYPHEN [19] and have a higher frequency in cases compared to controls, with the association of the SNP at position 158590601 reaching statistical significance (OR = 4.475, CI = 1.24–16.12, Chi^2^ = 6.28, p = 0.012). No association was found with age of onset (data not shown). This significantly associated rare SNP was genotyped in additional samples by individual genotyping. In total 2016 samples were genotyped comprising AD and control individuals as shown in table 3. There is a much smaller effect size in the UK population which had a lower MAF, where the association would not reach statistical significance alone. There was no association identified in the remaining samples from other Western European populations. Overall there was a weakly significant effect in a Meta-analysis of all the samples studied (table 3, using random effects model z = 1.98, p = 0.048).

**Table 2:** Association analysis for validated SNPs.

Position (hg18, Mar. 2006)	Amino Acid Change	Case			Control			chi square P value (1 df)
		N	Homozygous for Minor Allele	heterozygous	N	homozygous for Minor allele	heterozygous	
158588123	D249D	276	0	20	344	1	31	0.31
158588214	T280P	280	0	1	341	0	0	0.97
158590601	N417Y	287	0	11	345	0	3	0.012
158592193		288	0	1	345	0	0	0.96
158594972		288	0	0	345	0	1	0.84

**Table 3:** Genotyping information for the meta-analysis of the effect of the SNP at position 158590601 (N417Y) in all cohorts.

Study*	N AD cases	N controls	N417Y FrequencyCase	N417Y FrequencyControl	OR	95% CI	% Weight
Greece	347	369	0.016	0.004	3.994	1.105–14.439	42.14
UK	421	593	0.007	0.004	1.700	0.515–5.608	48.86
Other Western Europeans	145	141	0.003	0.004	0.979	0.061–15.807	9.00
D+L pooled OR					2.319	1.007–5.340	100.00

*The Greek samples comprise of the pooled sample individual genotyping results, with some additional individuals recruited in the same cohort. The UK and “Other Western European samples” are from the MRC cohort, and the Addneuromed study. The UK samples comprise of individuals recruited from centres in London and Cardiff, and the “Other Western Europeans” from centres in Italy, Poland, France and Finland (see methods and references for more details).

## Discussion

We demonstrate along with other recent publications [20]–[22] that it is possible to use pooled samples to screen for rare SNPs in large pools and after quality control obtain reliable allele frequency data. Although the cost of next-generation sequencing is reducing, it is still expensive and time consuming to resequence large numbers of individuals. This technique makes the large sample sizes required to have enough power to detect association more affordable.

In studies with a high depth of coverage it is the sequencing error rate (both sequencing and PCR errors) that is the limiting factor in the identification of very rare variants. An inadequacy in this study is that a single heterozygote SNP in one individual in the pool would have an allele frequency below that of some sequencing errors. We were able to identify variants as low as one individual in the pool, but we cannot rule out that some true variants have not been identified. A reduction in pool size could insure that the lowest frequency SNP would have an allele frequency above that of the distribution of sequencing error.

Limitations to the pooling approach are difficulties in ensuring equimolar representations of samples. In this study careful quantification of individual DNA samples and accurate combining has provided accurate allele calling, as shown by the strong correlation of allele frequencies obtained with pooled sequencing compared to individual genotyping. Increased accuracy in sample representation, and allele frequency estimation may have been achieved by performing separate PCR reactions in each individual and pooling of PCR products [22], although this would have resulted in increased costs and DNA use. A limitation of the use of pools is the loss of individual genotype data. For example one cannot test for an association under different models, or look at gene-gene or gene-environment interactions. An alternative approach gaining popularity is the use indexed libraries [3], [23], [24] and combinational pooling strategies [25], [26]. Although at present there are limitations to the number of samples that can feasibly be pooled, taking into account the cost and time constraints of preparing a library for each sample. We have shown that pooling before library generation without indexing is a viable alternative, saving cost and time and allowing the pooling of many more individuals, which is an especially useful way of screening large numbers of samples in a relatively small region. With recent improvements in read length, and sequence output as well as improving allele calling algorithms especially designed for pooled analysis the capacity of this technique is growing [27]–[30].

Out of 16 SNPs identified from pooled sequencing 11 were genotyped in individual samples and of these six were validated. There was an excellent correlation between the sequencing and individual genotyping results, but only for samples that had a forward to reverse read ratio score of less that 1. This indicates that the DNA pooling step was very accurate, but because of bias introduced in the sequencing (most likely at the PCR amplification stages) single genotyping validation is required for all but allele frequencies passing the most stringent quality control thresholds.

There are no common SNPs previously identified in Caucasian individuals in the regions sequenced as listed in dbSNP (build 130). The regions were investigated in the publically available SNP call data from the pilot 1000 genomes project data (www.1000genomes.org). Pilot 1 contains low depth whole genome sequencing of 60 individuals. Pilot 3 contains high depth sequencing of around 1000 gene regions in which the regions studied here are represented. The only SNPs that were identified in both the 1000 genomes data and our data are the SNPs at positions 158588123 and 158587689 which both have a minor allele frequency of 0.035 in the case pool and 0.048 in the control pool. In both pilot 1 and pilot 3 these SNPs have an allele frequency of 0.04. No other SNP above a frequency of 0.01 was identified in the 1000 genome data which were not identified in our sample. This is encouraging as it suggests that any SNP of a reasonable frequency had been detected. It is as expected that very rare SNPs identified are likely to be population specific.

Association analysis was carried out on the identified variants. There was no significant effect of the more common variants (at positions 158588123 and 158587689). The rare variants were also tested for association although in this sample size there is limited power to detect an association of variants with low MAF. The two nonsynonomous SNPs were found to be over represented in the case pool compared to the control pool with the N417Y variant reaching a level of statistical significance. The N417Y residue is located in the ectodomain of the protein. Examination of residues 261–502 reveal sequence similarity to the peptidase family [31]. The region has been shown to act as a recognition site for γ-secretase with the major substrate recognition site consisting of Glu333 and its nearby residues. Although it is conceivable that sequences outside residues 312–340 may contribute to substrate binding [31]. Deep alignment of mammalian species shows little conservation at this residue, but this particular amino acid change is not seen in any other species suggesting it may not be well tolerated. The substitution remains as a non-charged polar amino acid, but the change results in a large size difference. It is also a putative glycosylation site, suggesting there is still potential for an effect on the protein function. This variant has been previously identified in two genetic screens. Dermaut et al [10] identified the substitution in one individual from a screen of 78 Dutch Familial early onset AD cases (FAD). A follow up in a larger number of 354 FAD and 475 controls of Italian origin found an equal frequency in both. Additionally Confaloni et al [17] screened 174 sporadic AD patients and found two individuals carrying this mutation. It was also identified in one out of 191 controls (although a relatively young control), therefore showing a similar allele frequency as in our case and control groups. Functional analysis has been carried out on the substitution N417Y [10]. Aβ40 and Aβ42 levels were measured in conditioned media of HEK-293 cells transfected with the mutant and wild-type Nicastrin cDNA. Results were compared with known clinical mutations that cause FAD. Unlike the effects of the causal FAD mutations Aβ40 and Aβ42 levels were similar for N417Y and wild type NCSTN. These results do not disprove a functional effect of the substitution as it may be that the effect is modest or only apparent in vivo. The SNP was genotyped in a larger sample size of sporadic AD and control individuals (table 3). No significant effect was seen in these populations, but in a meta analysis including the Greek samples there is a significant association. As with many rare variants there is likely to be population heterogeneity, in terms of different allele frequencies, and different genetic background between populations.

In conclusion we have screened the coding regions of the Nicastrin gene and identified rare variation. We did not identify any novel rare variants that are likely to have an effect on the protein function. But we did find a modest association with a previously identified non-synonymous SNP, although this effect was not replicated in a larger sample size comprising other European populations. The present study validates the use of pooled DNA in the sequencing of small regions of interest as a screening method for the identification of rare SNPs.

## Materials and Methods

### Ethics Statement

Ethical approval for the main sample was obtained the Ethics Committee of “George Papanicolaou” Hospital, Exohi Thessaloniki.

For the follow-up genotyping ethical approval was obtained from the local ethics committee at each site. For the AddNeuromed project: Poland; the Ethics Committee of the Medical University of Lodz, Finland; The Ethical Committee of Kuopio University Hospital, Italy; Comitato Etico Aziende Sanitarie – Umbria, and France; CPP Midi Pyrennees (Comite Protection des Personnes). For the Medical Research Council Genetic Resource for Late-onset AD samples ethical approval was obtained from REC (Research Ethics Committee) for Wales. Informed consent was obtained from all participants in writing. For Alzheimer's patients this consent was given by their next of kin or carer.

### DNA Samples, Pooling and PCR

A cohort of Greek blood samples was collected from clinics at Thessaloniki hospital (Aristotle University, Thessaloniki, Greece) from Caucasian patients and their carer (if unrelated i.e. husband/wife for age matched control). Subjects were assessed using the Mental State Examination (MMSE) [32] and diagnosed according to the criteria of the National Institute of Neurological and Communication Disorders and Stroke and the Alzheimer's Disease and Related Disorders Associations (NINCDS-ADRDA) [33]. Controls were also recruited from the Greek blood donation service. Local hospital ethical approval was obtained for all samples collected.

Genomic DNA was extracted from whole blood using a method based on an organic deproteinization reagent. Pools of case and control DNA were constructed with quantification of each sample using fluorometry in triplicate (using Quant-iT PicoGreen® dsDNA kit (Invitrogen) and the fluroskan Ascent FL fluorometer). All pipetting steps were carried out by hand with a volume of 5 µl or greater to ensure accuracy. The two closest of the three readings were selected and an average taken. The concentration was only accepted if the difference between the two readings was less than 5% of the average concentration. 311 AD cases (with age of onset greater than 60 years) and 360 controls were used to produce the pools. 16 oligonucleotides primer pairs were designed to cover the 17 exons of the gene and the regions amplified from the pooled DNA using standard PCR procedures (table S1 contains the primer sequences). Reactions were carried out in 50 µl volumes using 25 ng of pooled DNA for both cases and controls. 2 µl of each PCR product was electrophoresed and visualised using SYBR green (Roche). PCR product concentration was estimated by comparison of the level of fluorescence to a serial dilution of Generuler DNA ladder (Fermentas Life Sciences) using image J software [34]. The PCR products were then combined in 2 case and control pools. The volume added was adjusted according to the size of the PCR product ensuring equal molar concentration.

Replication genotyping was performed using samples obtained though AddNeuromed, a cross European, public/private consortium developed for AD biomarker discovery [35] and a UK cohort from the Medical Research Council Genetic Resource for Late-onset AD [36].

### Library preparation and sequencing

The PCR products were extracted using the QIAquick PCR Purification Kit (Qiagen) as per manufactures' instructions. End repair was carried out using End-it DNA Kit (Cambio) to convert the DNA ends to 5′-phosphorylated blunt-end DNA. Extraction was again carried out using the QIAquick PCR Purification Kit and concatermerisation of the repaired PCR products was carried out using the Quick Ligation kit (NEB). The samples were fragmented by nebulisation from which a library was prepared according to the Illumina protocol. The libraries were run on the Illumina Genome Analyzer II for 36 cycles. The reference genome used for sequence alignment was the Human Build 36.1 finished human genome assembly (hg18, Mar. 2006). The images were analyzed with the pipeline software (version 1.0, Illumina software) to undertake base calling and sequence alignment to the reference genome. The ELAND algorithm was used with the ‘ELAND extended’ option.

### Data Analysis

MAQ [37] was used to align the short read sequence data obtained to a reference sequence using the default parameter of only considering positions that have two or fewer mismatches in the first 28 bp. For mismatches the maximum sum of mismatching bases quality score of 70 is used as a cut off for alignment. The sequence alignment outputs were converted to a “pileup” format from which allele frequencies were elucidated. After excluding the primer sequences the frequency of each substitution was plotted as a histogram to access the distribution of errors. We assumed an error model similar to that of Out et al, [22] where errors occur at a constant proportion estimated by a Poisson distribution and took the same conservative value of the error rate. The 97.5% percentile of the observed distributions for each base change was therefore taken as the cut off for variant calling. Forward and reverse strands were considered separately and variants were only considered if identified in both above the threshold.

Statistical analysis was carried out using STATA.

### Genotyping

Single nucleotide polymorphisms (SNPs) were genotyped using the Sequenom® MassARRAY technology (Sequenom®; San Diego, CA, USA). Additionally one SNP was genotyped in a larger sample size using TaqMan Single Nucleotide Polymorphism (SNP) Genotyping Assay (Applied Bio systems). SNP-specific primers and probes were designed and assays were performed according to the manufacturer's instructions.

## Supporting Information

Table S1 Region-specific primers for amplification of 16 exons in the Nicastrin gene.
(DOC)

## References

[1] Harold D, Abraham R, Hollingworth P, Sims R, Gerrish A (2009). Genome-wide association study identifies variants at CLU and PICALM associated with Alzheimer's disease.. Nat Genet.

[2] Lambert JC, Heath S, Even G, Campion D, Sleegers K (2009). Genome-wide association study identifies variants at CLU and CR1 associated with Alzheimer's disease.. Nat Genet.

[3] Mitsui J, Fukuda Y, Azuma K, Tozaki H, Ishiura H (2010). Multiplexed resequencing analysis to identify rare variants in pooled DNA with barcode indexing using next-generation sequencer.. J Hum Genet.

[4] Sidransky E, Nalls MA, Aasly JO, Aharon-Peretz J, Annesi G (2009). Multicenter analysis of glucocerebrosidase mutations in Parkinson's disease.. N Engl J Med.

[5] Butler AW, Ng MY, Hamshere ML, Forabosco P, Wroe R (2009). Meta-analysis of linkage studies for Alzheimer's disease–a web resource.. Neurobiol Aging.

[6] Dries DR, Yu G (2008). Assembly, maturation, and trafficking of the gamma-secretase complex in Alzheimer's disease.. Curr Alzheimer Res.

[7] Levy-Lahad E, Wasco W, Poorkaj P, Romano DM, Oshima J (1995). Candidate gene for the chromosome 1 familial Alzheimer's disease locus.. Science.

[8] Rogaev EI, Sherrington R, Rogaeva EA, Levesque G, Ikeda M (1995). Familial Alzheimer's disease in kindreds with missense mutations in a gene on chromosome 1 related to the Alzheimer's disease type 3 gene.. Nature.

[9] Yu G, Nishimura M, Arawaka S, Levitan D, Zhang L (2000). Nicastrin modulates presenilin-mediated notch/glp-1 signal transduction and betaAPP processing.. Nature.

[10] Dermaut B, Theuns J, Sleegers K, Hasegawa H, Van den BM (2002). The gene encoding nicastrin, a major gamma-secretase component, modifies risk for familial early-onset Alzheimer disease in a Dutch population-based sample.. Am J Hum Genet.

[11] Cousin E, Hannequin D, Mace S, Dubois B, Ricard S (2003). No replication of the association between the Nicastrin gene and familial early-onset Alzheimer's disease.. Neurosci Lett.

[12] Helisalmi S, Dermaut B, Hiltunen M, Mannermaa A, Van den BM (2004). Possible association of nicastrin polymorphisms and Alzheimer disease in the Finnish population.. Neurology.

[13] Deary IJ, Hamilton G, Hayward C, Whalley LJ, Powell J (2005). Nicastrin gene polymorphisms, cognitive ability level and cognitive ageing.. Neurosci Lett.

[14] Ma Z, Han D, Zuo X, Wang F, Jia J (2009). Association between promoter polymorphisms of the nicastrin gene and sporadic Alzheimer's disease in North Chinese Han population.. Neurosci Lett.

[15] Orlacchio A, Kawarai T, Polidoro M, Paterson AD, Rogaeva E (2004). Lack of association between Alzheimer's disease and the promoter region polymorphisms of the nicastrin gene.. Neurosci Lett.

[16] Zhong L, Dong-hai Q, Hong-ying L, Qing-feng L (2009). Analysis of the nicastrin promoter rs10752637 polymorphism and its association with Alzheimer's disease.. Eur J Neurosci.

[17] Confaloni A, Terreni L, Piscopo P, Crestini A, Campeggi LM (2003). Nicastrin gene in familial and sporadic Alzheimer's disease.. Neurosci Lett.

[18] Wang B, Yang W, Wen W, Sun J, Su B (2010). Gamma-secretase gene mutations in familial acne inversa.. Science.

[19] Sunyaev S, Ramensky V, Koch I, Lathe W, Kondrashov AS (2001). Prediction of deleterious human alleles.. Hum Mol Genet.

[20] Druley TE, Vallania FL, Wegner DJ, Varley KE, Knowles OL (2009). Quantification of rare allelic variants from pooled genomic DNA.. Nat Methods.

[21] Nejentsev S, Walker N, Riches D, Egholm M, Todd JA (2009). Rare variants of IFIH1, a gene implicated in antiviral responses, protect against type 1 diabetes.. Science.

[22] Out AA, van Minderhout IJ, Goeman JJ, Ariyurek Y, Ossowski S (2009). Deep sequencing to reveal new variants in pooled DNA samples.. Hum Mutat.

[23] Craig DW, Pearson JV, Szelinger S, Sekar A, Redman M (2008). Identification of genetic variants using bar-coded multiplexed sequencing.. Nat Methods.

[24] Cronn R, Liston A, Parks M, Gernandt DS, Shen R (2008). Multiplex sequencing of plant chloroplast genomes using Solexa sequencing-by-synthesis technology.. Nucleic Acids Res.

[25] Erlich Y, Chang K, Gordon A, Ronen R, Navon O (2009). DNA Sudoku–harnessing high-throughput sequencing for multiplexed specimen analysis.. Genome Res.

[26] Prabhu S, Pe'er I (2009). Overlapping pools for high-throughput targeted resequencing.. Genome Res.

[27] Druley TE, Vallania FL, Wegner DJ, Varley KE, Knowles OL (2009). Quantification of rare allelic variants from pooled genomic DNA.. Nat Methods.

[28] Wang T, Lin CY, Rohan TE, Ye K (2010). Resequencing of pooled DNA for detecting disease associations with rare variants.. Genet Epidemiol.

[29] Bansal V (2010). A statistical method for the detection of variants from next-generation resequencing of DNA pools.. Bioinformatics.

[30] Koboldt DC, Chen K, Wylie T, Larson DE, McLellan MD (2009). VarScan: variant detection in massively parallel sequencing of individual and pooled samples.. Bioinformatics.

[31] Shah S, Lee SF, Tabuchi K, Hao YH, Yu C (2005). Nicastrin functions as a gamma-secretase-substrate receptor.. Cell.

[32] Folstein MF, Folstein SE, McHugh PR (1975). “Mini-mental state”. A practical method for grading the cognitive state of patients for the clinician.. J Psychiatr Res.

[33] McKhann G, Drachman D, Folstein M, Katzman R, Price D (1984). Clinical diagnosis of Alzheimer's disease: report of the NINCDS-ADRDA Work Group under the auspices of Department of Health and Human Services Task Force on Alzheimer's Disease.. Neurology.

[34] Abramoff MD, Magelhaes PJ, Ram SJ (2004). Image Processing with ImageJ.. Biophotonics International.

[35] Lovestone S, Francis P, Kloszewska I, Mecocci P, Simmons A AddNeuroMed–the European collaboration for the discovery of novel biomarkers for Alzheimer's disease.. Ann N Y Acad Sci.

[36] Proitsi P, Hamilton G, Tsolaki M, Lupton M, Daniilidou M (2009). A Multiple Indicators Multiple Causes (MIMIC) model of Behavioural and Psychological Symptoms in Dementia (BPSD).. Neurobiol Aging.

[37] Li H, Ruan J, Durbin R (2008). Mapping short DNA sequencing reads and calling variants using mapping quality scores.. Genome Res.

