# Supplementary material for: Deep Sequencing of the Nicastrin Gene in Pooled DNA, the Identification of Genetic Variants That Affect Risk of Alzheimer's Disease
Source: PLoS One. 2011 Feb 25;6(2):e17298. doi: 10.1371/journal.pone.0017298 (PMC3045431; doi:10.1371/journal.pone.0017298)
Supplement: Table S1 — Region-specific primers for amplification of 16 exons in the Nicastrin gene. (DOC) [file pone.0017298.s001.doc]

| Exon | Forward primer | Reverse primer | PCR Product (bp) |
| --- | --- | --- | --- |
| 1 | aaacacgaacttccggtctc | cctgctagtaggacgcagaaa | 203 |
| 2 | cacgatagaagctagtacctgtgtc | caatgtccacaaacatgaatcc | 201 |
| 3 | gggcaacagcttttcagttc | gctctccagcagaaccatgt | 265 |
| 4 | tccttcatggaattcctttcc | agaccgaagcaacagtgagc | 213 |
| 5 | agtccccctattccccatc | attgctaggagctggcagtg | 203 |
| 6 | cctcctccttctgggatgta | ttgatgctgaaggtgctttg | 221 |
| 7 | ggcactggtcagagatttcc | gggcctaccagctctttttg | 258 |
| 8 | ggagcaagaaaggaggtttg | agcccttaccccttgaaaga | 232 |
| 9 | ttgaggtgacctgagatagtcg | acatacctgtcccagctcca | 228 |
| 10 | cggtcttaactgcaggaacc | aggaaatgaggggagatgct | 213 |
| 11 | ggccacattggagaagagtg | cagtgaccttcctcccagac | 234 |
| 12 & 13 | cactgccaaggtagcactga | acactagggcaagggaggat | 284 |
| 14 | gccagctactgtctcccaac | cctgcctgaggatagactgg | 205 |
| 15 | acccaccaaatcttcccttc | caccaccactcacatccttg | 206 |
| 16 | ccttgtctcctgcctttgaa | acagatccctgccacctatg | 158 |
| 17 | agcttttcttgccagctcag | gtcctcaggggctcaatgta | 439 |
